# Reframing the lived experience of persistent dizziness: insights from active inference

**DOI:** 10.3389/fpsyg.2026.1867836

**Published:** 2026-10-14

**Authors:** Marc A. Broberg, Mick Thacker, Karl J. Friston, Jorge E. Esteves

**Affiliations:** 1 Two Trees Physical Therapy, Ventura, CA, United States; 2 Royal College of Surgeons in Ireland, Dublin, Ireland; 3 Health Sciences University Centre for PAIn Research, Bournemouth, United Kingdom; 4 Wellcome Trust Centre for Neuroimaging, Institute of Neurology, University College London, London, United Kingdom; 5 Escola Superior de Saúde Atlântica, Barcarena, Portugal

**Keywords:** active inference, enactivism / ecological psychology, free energy principle (FEP), lived experience, persistent dizziness, phenomenology, PPPD, ViolEx model

## Abstract

We present an active inference account of persistent dizziness predicated on models of enactivism and ecological psychology; including first-hand accounts of the patient’s lived experience. Mechanistically, we consider persistent dizziness to reflect an initial failure of sensory attenuation of vestibular and other sensory prediction errors. This failure becomes self-maintaining if the patient infers—or learns—that unattenuated prediction errors cannot be resolved through action. The ensuing formulation provides an integrative account of the pathophysiology of dizziness and how it underwrites—and is maintained by—affective inference and an altered sense of agency that shapes the experience of the person with dizziness. We foreground the holistic person-centered approach on offer from a clinical perspective, and consider complementary levels of therapeutic intervention under this theoretical framework.

## Introduction

Nearly 20% of the population will experience dizziness severe enough to lead to medical consultation, sick leave, or disruption of daily activities at some point in their lives (Neuhauser, 2016). The initial etiology of dizziness can be vestibular, medical, or anxiogenic in nature (Staab, 2023; Staab et al., 2017). Approximately 25% of individuals with acute dizziness across neuro-otologic triggers will have symptoms that extend beyond the expected recovery timeline of 3 months (Staab et al., 2017), often despite evidence of compensation for the initial cause (Cousins et al., 2017; Staab et al., 2017; Halmagyi et al., 2010). Of the potential acute etiologies, acute vestibular neuritis is the most studied, with approximately 20–50% of individuals affected going on to experience persistent symptoms (Godemann et al., 2004; Godemann et al., 2005; Lee and Kim, 2020; Adamec et al., 2014; Bergenius and Perols, 1999). Reports of persistent dizziness triggered by BPPV, the most common acute neuro-otologic condition, range from approximately 8 to 32% (Cha et al., 2017; Heinrichs et al., 2007). Following vestibular ablation for intractable Ménière’s Disease, 50% of patients reported ongoing imbalance beyond 1 year (Reid et al., 1996), with 10–15% experiencing constant symptoms (Halmagyi et al., 2010; Reid et al., 1996).

Furthermore, many individuals living with persistent dizziness feel that they are unable to return to work and social activities long-term (Huppert et al., 2005) and report low subjective wellbeing (Tschan et al., 2011). Anxiety and depression are commonly associated with persistent dizziness and have been implicated as both causes and consequences of ongoing symptoms (Cousins et al., 2017; Bronstein and Dieterich, 2019; Trinidade et al., 2023; Dieterich and Staab, 2017; Mahoney et al., 2013). Taken together, persistent dizziness is closely associated with reduced quality of life, significant psychological distress, psychiatric comorbidity, avoidance behaviors, and complex, multifactorial symptom presentations (Alahmari and Alshehri, 2025; Kristiansen et al., 2019; Lotze et al., 2025).

Early conceptual models of persistent dizziness focused on the psychological factors involved in triggering and sustaining the condition (Brandt, 1996; Staab, 2013; Dieterich and Staab, 2017). Treatments based on these models were based on cognitive behavioral therapy (CBT) interventions designed to treat psychological conditions, specifically panic disorders (Mahoney et al., 2013; Edelman et al., 2012). Subsequently, “CBT-informed” approaches were adopted by rehabilitation professionals and delivered in conjunction with traditional vestibular rehabilitation (VR) exercises (Herdman et al., 2022). More recently, a shift in emphasis toward a more encompassing framework of persistent dizziness, extending beyond psychological factors, has occurred, along with updated diagnostic guidelines, yielding “persistent postural perceptual dizziness (PPPD)” (Staab et al., 2017). For the purposes of this paper, we refer to “persistent dizziness” with the intention of unifying and simplifying dialog around any condition involving ongoing dizziness (including PPPD).^1^ Conceptual models of PPPD consider the role of agency (Staab, 2023; Arshad et al., 2022), cognition (Staab, 2023; Arshad et al., 2022), hierarchical processing (Staab, 2023; Arshad et al., 2022), attention (Staab, 2023; Arshad et al., 2022), spatial orientation (Staab, 2023), and locomotion (Staab, 2023). The most recent model iteration (Staab, 2023) intends to integrate and further develop the frameworks of its predecessors and will serve as the foundation we will consider and extend. In this pathophysiological model, a person with a precipitating (acute) dizziness episode experiences heightened perception of motion, visual dependence, and high-risk (constrained) postural, gaze, and movement patterns, which—accompanied by anxiety and hypervigilance—do not return to pre-symptom levels. According to this model, based on optimal control theory, persistent misperception of motion results in cost function optimization (i.e., over-prioritization) of stability, which is associated with altered functional connectivity in multimodal sensorimotor (i.e., vestibular cortex) and broader supraspinal spatial navigation networks (i.e., hippocampus, frontal, parietal regions; Indovina et al., 2021), contributing to ongoing symptoms and constrained motor behaviors (Staab, 2023). While this model offers valuable insights into the multiple factors involved in persistent dizziness, it has not, to our knowledge, informed or motivated novel care strategies beyond its predecessors. This may be because pathophysiological models do not offer data that guide clinical engagement, which ideally includes interactive dialog exploring what the experience has been like for the person (Dierckxsens, 2025); in other words, there is a disconnect between the lived experience of the person and the pathophysiological models that attempt to explain the condition. Moreover, pathophysiological models do not fully explain motivational factors involved in (for example) avoidant behaviors. A phenomenological extension of the current model, i.e., one that seeks to understand and further elaborate the lived experience of the person with persistent dizziness, therefore offers a clinically oriented interpretation that informs clinical engagement.

Narrative themes emerging from qualitative and phenomenological studies of this lived experience include: disrupted agency (Gamble et al., 2023; Sezier et al., 2019; Olsson Möller et al., 2014); invalidation by healthcare providers and social others (Gamble et al., 2023; Sezier et al., 2019; Kruschinski et al., 2010; Mendel et al., 1997); difficulties interpreting symptoms (Kruschinski et al., 2010; Mendel et al., 1997); uncertainty about the future (Sezier et al., 2019; Olsson Möller et al., 2014); emotional distress (Gamble et al., 2023; Sezier et al., 2019; Mendel et al., 1997); and a persistent struggle for control and coherence (Olsson Möller et al., 2014; Mendel et al., 1997). Although increasingly recognized as vital for informing person-centered care (Beames et al., 2021), such first-person accounts of lived experiences are underrepresented in the clinical literature at large, and for persistent dizziness in particular (Gamble et al., 2023). Moreover, to our knowledge, no existing conceptual model has incorporated the lived experience of individuals with dizziness into its framework. We therefore present an overarching framework—sufficiently expansive to incorporate these phenomenological perspectives within contemporary models—with implications for a clinically oriented and accessible account of persistent dizziness. The proposed mechanistic account is informed by themes espoused within enactivism and ecological psychology. Furthermore, enactivism and ecological psychology approaches are compatible with active inference—a corollary of the Free Energy Principle—which we apply to persistent dizziness for a deeper understanding of its phenomenology, with further insights into therapeutic targets for recovery. Additionally, active inference offers a biologically plausible process theory for addressing the theoretical limitations of optimal control theory (Friston, 2011), thereby providing a principled account of recovery that can be readily applied to the neurobiology and phenomenology of dizziness. In the remaining sections, we propose an extension of the current theoretical and clinical framework for persistent dizziness with the following commitments:

- Enactivism and Ecological Psychology offer a relatable and clinically accessible framework for conceptualizing persistent dizziness, which accommodates lived experiences.
- Active Inference provides a principled and biologically plausible process by which the above framework can be used to furnish an integrative understanding of the mechanisms and phenomena of persistent dizziness, thereby informing innovative approaches to education, research, and, most importantly, care.

## Enactivism

### Background / embodied cognition

Emerging from the concept of embodied cognition (Varela et al., 1991), Enactivism casts a person as an agent engaged in an active sense-making process, as described by a “4E approach” (Menary, 2010): *embodied, embedded, enacted, extended*, and (more recently) *emotive* as the “5th E” (Stilwell and Harman, 2019). An orientation rooted in phenomenology, which emphasizes the primacy of lived, first-person experience, is fundamental to enactivist theory. This theory posits that sense-making arises from the relational, embodied interaction of an agent with their environment, rather than solely from internal representational processes (Varela et al., 1991; Thompson, 2010; Gallagher and Zahavi, 2020). This theoretical stance has been applied to clinical settings, particularly through enactive frameworks for understanding and treating persistent pain. These frameworks reconceptualize pain not as a localized pathological signal but as a dynamic, multicausal, and embodied process influenced by affordances, expectations, and the agent’s situated action possibilities (Stilwell and Harman, 2019; Cormack et al., 2023; Vaz et al., 2023). An example of the dynamic, multidimensional nature of pain is reflected in how clinical interactions and contextual cues can influence the intensity of pain and the perceived outcome expectations, either positively or negatively (Zamfira et al., 2026; Kim et al., 2022; Howick et al., 2018; Testa and Rossettini, 2016). Given the significant phenomenological, biopsychosocial, and conceptual similarities between persistent pain and persistent dizziness, including shared experiences of disrupted agency, threat appraisal, and embodied uncertainty, an enactive framework not only aligns intuitively with dizziness but also provides a principled theoretical foundation for its clinical reconceptualization (Broberg and Boyd, 2023).

### 4E (enactive) approach to sense-making

#### Embodiment

Phenomenology offers a foundational framework for understanding embodiment, particularly through the distinction between the *lived body* (body-as-subject) and the *objective body* (body-as-object). Central to phenomenological thought is the claim that lived experience is indispensable for comprehending any condition, as experience is always mediated through the body—not merely as a biological entity, but as the center of perception, subjectivity, and meaningful engagement with the world (Boge-Olsnes et al., 2023; Mitchell and Meehan, 2022; Piran, 2017).

For example, in a video-based phenomenological study of women with chronic pelvic pain undertaking a standardized physiotherapy movement test, it was reported that participants were observed to play down their pain verbally while expressing it through breath-holding, muscular tension, grimacing and compensatory movement. When reviewing the recordings, they described their tense gait as a way of guarding against vulnerability. After treatment, one participant described being able to relinquish some of the control she had exerted over her movements and to feel freer (Boge-Olsnes et al., 2023).

Among people living with schizophrenia, absorbed, hands-on art-making shifted participants away from worry and reflective thought towards pre-reflective bodily engagement. One participant’s painting of his cat appeared to draw on the kinaesthetic memory of stroking it. Participants also described feeling at home while drawing, in contrast to the estranged, unhomelike world that can accompany the condition (Mitchell and Meehan, 2022). Piran’s (2017) Developmental Theory of Embodiment, derived from the life histories of girls and women, likewise understands embodiment as lived engagement with the world. This engagement is shaped through physical, mental and social-power pathways that can support freedom, agency and connection with the body, or constrain these possibilities.

These accounts resonate with the lived experience of persistent dizziness, in which people describe disrupted agency, a persistent struggle for control and invalidation by others (Gamble et al., 2023; Olsson Möller et al., 2014; Sezier et al., 2019). The accounts of guarded movement also offer a point of connection with the over-controlled postural strategies commonly reported in persistent dizziness (Staab, 2023).

The *lived body* (*Leib*) refers to the body as subject—experienced from within, in a first-person perspective—as the coenaesthetic ground of self-awareness, perception, emotion, action, and thought. It is inseparable from consciousness and foundational to the sense of being an embodied agent (Northoff and Stanghellini, 2016; Parnas and Handest, 2003; Mitchell and Meehan, 2022; Castellini et al., 2022). In contrast, the *objective body* (*Körper*) is perceived from the outside, as an observable, measurable object within biomedical and scientific frameworks—understood primarily through visual inspection, anatomical structure, and physiological function (Northoff and Stanghellini, 2016; Parnas and Handest, 2003; Castellini et al., 2022). Biomedical models largely emphasize this objective body, often at the expense of the subjective, lived experience that phenomenological approaches place at the forefront (Springmann et al., 2020). While objective models yield critical insights into biological mechanisms, phenomenological approaches centered on the body-as-subject reveal dimensions of experience that are person-centered, empathic, and clinically meaningful (Gallagher, 2023). In the context of persistent dizziness, an embodied approach bridges the *felt*, symptomatic experience—described by patients as feeling “off,” “wobbly,” “foggy,” or “nervous”—with the measurable, objective body, offering a more integrated and validating pathway for interpreting symptoms.

Advancements in neuroimaging have yielded insights into potential “brain correlates” of dizziness, i.e., changes in multimodal vestibular cortex (Cao et al., 2022; Indovina et al., 2021; Storm et al., 2025), hippocampus, frontal, parietal regions (Indovina et al., 2021), licensed by the position that brain regions potentially contain representational content encoding sensory (internal) and environmental (external) states (Gładziejewski, 2016). Interestingly, when galvanic vestibular stimulation was used to induce perceptions of self-motion, participants with dizziness reported greater perceptual responses than non-dizzy controls, which were proportional to the measured increases in multimodal vestibular cortex, consistent with sensory-neural amplification (Storm et al., 2025). This may reflect prior expectations rather than a neural signature of the condition as both perceptual and neural responses are biased by prior expectations and/or attention (Summerfield and De Lange, 2014) similar to responses seen in pain (Atlas and Wager, 2012). This alternate view is consistent with our current framing.

“Strong embodiment” positions maintain that embodied action within the world brings about meaning without the need for internal structural representations of sensory experiences or the environment (Sanches de Oliveira et al., 2021) and would endorse the argument that the brain activity encodes or reflects no such representational content (Brette, 2019).^2^ Without defending (or refuting) radical Enactivism, we assert that looking beyond a neurocentric framework, embodiment extends clinical dialogs, treatments, and research hypotheses to be considered within the context the lived body, which is experiential, relational, and real, rather than representational and ethereal.

The orientation of the body itself contributes substantially to the dizziness experience (e.g., upright vs. lying down postures). An embodied approach emphasizes practical interventions involving “the body” and its preeminent role in balance, mobility, and spatial tasks, promoting active (tactile) strategies that employ a more complete, first-person experience. Such an embodied (body-focused) approach can help restore a sense of agency, defined as feeling in control over one’s bodily actions (Haggard, 2017), which is often disrupted in a person with dizziness (Gamble et al., 2023; Sezier et al., 2019; Olsson Möller et al., 2014; Kruschinski et al., 2010; Mendel et al., 1997). Disrupted agency for a person with persistent dizziness can be described as a disembodiment or depersonalization experience, in which the person is not fully in control of their own thoughts, perceptions, and actions, further validating a body-focused approach (Ciaunica et al., 2022).

#### Embedded

The sense-making process is undertaken in an environment wherein the embodied agent is also embedded or situated (Gallagher, 2023). Indeed, our sensorium is attuned to our *‘lived world,’* (Merleau-Ponty et al., 2013) or niche-environment that suits our specific needs as organisms: e.g., (Gibson, 1979). Examples include our implicit encoding of gravity and temperature, which allows us to thrive in our environment. In the context of persistent dizziness, interactions within the environment are relevant, if not deterministic, to the patient’s experience. For example, busy environments with moving or visually complex stimuli can provoke symptoms in patients (Staab et al., 2017; Wilkins and Evans, 2010; Fernandez and Wilkins, 2008). Environments or situations that induce postural threats, such as standing at heights, are used experimentally to induce responses similar to persistent dizziness in participants without the condition (Cleworth and Carpenter, 2016; Naranjo et al., 2015; Zaback et al., 2016; Vermehren and Carpenter, 2020). Environments, including objects therein, also provide opportunities to reduce the burden of the experience (stabilizing oneself using a countertop or handrail—see affordances).

#### Extended

In an enactivist framework, cognition (sense-making) *extends* beyond the boundaries of the brain, body, and environment. Clark and Chalmers (1998) contend that a cognitive system consists of an active coupling of an agent to an external entity used in sense-making, citing examples of memory aids, written instructions, and language itself as being extensions of cognition and therefore mind. Kirchhoff and Kiverstein (2021), provide a formal account of the dynamics of the active coupling process, providing further evidence for—and boundaries of—extended cognition. Briefly, they argue that an agent’s cognition is bounded, yet dynamic in that it may extend into the world as needed to couple with resources that afford sense-making. Socially extended cognition, in which a collective, participatory sense-making process is realized through interactions between individuals or groups (Gallagher, 2013; Gallagher, 2023), provides an important context for a person with persistent dizziness. A person’s experience affects and is affected by interactions with other individuals, including family, peers, providers, and institutions, including employers and health care establishments. We propose the therapeutic alliance—the relationship formed between client and clinician with the intention of collaborating to produce positive health outcomes (Miciak et al., 2019; McParlin et al., 2022)—is a critical example of extended cognition (Mehl et al., 2020). While the therapeutic alliance provides an opportunity to form positive expectations for recovery (Howick et al., 2018), the person with dizziness is often misunderstood, or invalidated by others, including health care providers (Gamble et al., 2023). We will discuss the therapeutic alliance in greater detail in the following sections.

#### Enacted

Enactivists view perception and action as recurrent, codependent loops, in which perception guides action, which, in turn, constructs perception (Varela et al., 1991). Meaning is brought forth as the agent deploys multimodal actions that “palpate” the world. Moreover, agency itself is established as actions and their consequences (sensorimotor contingencies) are enacted and perceived as being caused by the “organism” (Buhrmann and Di Paolo, 2017). A person with dizziness *enacts* behaviors that reflect their felt experience, which in turn shapes what is felt. For example, when a person perceives a rocking sensation, a stiff postural strategy is assumed (Staab, 2023; Schniepp et al., 2013). The resultant altered sway pattern contributes to increased rocking frequency in the context of heightened perception (Staab, 2023; Krafczyk et al., 1999).

#### Emotive

In their work exploring the applications of enactivism as an approach for pain, Stilwell and Harman (2019) proposed a 5th E, *emotive*. Other Enactivists have positioned affect as intrinsically linked to the active sense-making process, and that motivational and affective features are integrated with (inseparable from) active engagement (Colombetti, 2017; Degenaar and O’Regan, 2017).

Affect is integral to the sense-making process in that it ascribes meaning (value) to the objects of perception. Thus, a glass of water is perceived to have affective value to the thirsty hiker, who is motivated to act, thereby preserving their homeostasis. This assumes that affect is broadly defined to include emotions, moods, and motivational states related to homeostasis, allostasis, and autonomy, such as desires, needs, fatigue, and pain (Colombetti, 2017). Moreover, affective valence is argued to represent one’s inference about their ability to successfully produce adaptive actions (Hesp et al., 2021). Therefore, affect (emotion) is not a distinct external characteristic of an experience. Rather, it is an intrinsic component of the sense-making process itself. The implications of this reframing can inform a holistic therapeutic approach that extends beyond addressing “emotional components” in isolation, recognizing they are an expressed reflection, scoring the person’s perceptions of their ability to successfully enact a plan that is consistent with their intentions.

## Ecological psychology

Based on the work of James Gibson, ecological psychology focuses on understanding perception and action as direct interactions between organisms and their environments (Segundo-Ortin, 2020). While some have highlighted a historical theoretical division between enactivism and ecological psychology (Heft, 2020), others have described a resonance between the two approaches (Ryan Jr and Gallagher, 2020; Segundo-Ortin, 2020); here we emphasize their compatibility and collective strength in framing and addressing the fundamental concerns (identified by phenomenology) of individuals with persistent dizziness. Enactivists and ecological psychologists are unified by shared views of embodiment (shared rejection of representationalism), perception-action-environmental linkages, and *affordances* (Segundo-Ortin, 2020). Enactivism and Ecological Psychology frameworks contextualize perception in terms of affordances, coupling the embodied agent’s perception-action cycles to the environment in which it is embedded.

Affordances are understood as the perceived actions available to an agent within an environment or the objects therein (Gibson, 1979). Elaborating on the example above, a glass of water is perceived as something which can be grasped and drunk from rather than a fluid-filled cylinder. In the context of persistent dizziness and (perceived) imbalance, a countertop provides stability when touched. *Attunement* applies one’s intentions and goal-directed actions to selectively engage with environmental affordances (Ryan Jr and Gallagher, 2020). Indeed, the sense of agency is fundamentally anchored to one’s ability to enact action plans that incorporate environmental affordances. Agentive behavior is reflected in resourceful engagement with the lived world, as individuals perceive, select, and act upon the possibilities their environment offers, which are shaped by their goals, values, and subjective interpretations (Han, 2019; Mutlu, 2021; Sharma-Brymer et al., 2024; Zhang et al., 2024). Taken together, enactivism and ecological psychology can offer a clinically accessible and cohesive framework linking the lived experience to intentional behaviors in light of one’s embodied, contextualized coupling to the (physical and social) environment. With the experience of persistent dizziness now holistically framed, a person-centered reference is more fully appreciated. This “packaging” reflects both the readiness and need for a process theory to unify and inform a framework for understanding and treating the person with persistent dizziness.

## (En)active inference

An enactive approach builds upon contemporary models of persistent dizziness by incorporating the lived experience of the person with dizziness, offering a more holistic and accessible clinical framework for understanding the individual with the condition. While this approach offers a unifying and holistic conceptual perspective on the condition, it does not provide a principled account that addresses the dynamics of how its constituent Es interact, nor does it model the actual recovery process. However, both enactivism and ecological psychology are compatible with—and formally elaborated under—an active inference framework (Ramstead et al., 2020; Kiverstein et al., 2019a; Kiverstein et al., 2019b; Veissière et al., 2020; Albarracin and Poirier, 2022; Bruineberg et al., 2018) which we will use to provide a principled account of the dynamics of persistent dizziness, including the potential mechanics of recovery, with implications for intervention.

Active inference is a process theory, based on the Free Energy Principle (FEP), which describes a biological organism as operating under the imperative to minimize free energy (an approximation of surprise),^3^ which is most intuitively regarded as (precision-weighted) prediction error. In this setting, the prediction error is simply the difference between an organism’s predictions and observations (i.e., sensory inputs; Friston, 2010; Friston et al., 2010). Active inference describes the process by which an organism’s actions serve to optimize sensory inputs to minimize prediction errors (Friston, 2010; Friston et al., 2010). Thus, active inference provides a unifying account of active sense-making (resolving uncertainty) through perception-action cycles, which function to minimize precision-weighted prediction errors (Friston, 2010; Friston et al., 2010). The precision here corresponds to the confidence or credence afforded prediction errors, such that if prediction errors are deemed to be very precise and reliable, they have a greater influence on sense making—and decision-making—by updating internal representations to provide better predictions to resolve prediction errors (and thereby reduce free energy). Consciously, precision weighting corresponds to attention, with the physiological mechanism understood as synaptic gain (Feldman and Friston, 2010).

In terms of enactivism and ecological psychology, active inference is an integrative framework for understanding the *dynamics* of an embodied, embedded agent—how internal (embodied) and external (embedding) states co-evolve over time. These dynamics are governed by the agent’s ability to predict the causes of its observations (sensations), through action and perception (Friston, 2010; Friston et al., 2010). This predictive ability depends on exploiting probabilistic relationships between observations (o) and states (s) of the world, specifically their joint probability, p(o,s). Statistically, p(o,s) is a probabilistic model that enables the agent to generate predictions—a generative model. More specifically, the generative model (GM) consists of *prior beliefs* about the “states (s)” of the world and the *likelihood* of state-dependent sensations, or “observations (o),” given their causes (Smith et al., 2022a):

GM=p(s,o)=p(s)⋅p(o│s)=p(o)⋅p(s│o)

Here, p(s) is the *prior probability* of a state (s) before making a (sensory) observation (o), and p(o│s) is the “*likelihood*” or probability (p) that one would make these observations (o), for a given (│) state. Therefore, the generative model is simply a probabilistic explanation of how unobservable (hidden) states of affairs cause observable sensations. Technically, “hidden” states (of the world) are not *directly* observable because they are behind the agent’s sensory veil,^4^ but they do generate observations (sensations) from which the agent infers their cause.

Given that Enactivists and Ecological Psychologists (largely) endorse the concept of (strong) embodiment that rejects representational “models,” it is important to clarify that the generative model is a statistical (probabilistic) model (with prior beliefs and likelihood mappings). This is a model of the relationship between hidden causes and consequent sensory impressions that enables inference to the best explanation about the causes of the sensorium. Crucially, sensory samples are generated through interaction with the world—in which the embodied agent is *embedded*—that is *enacted* through behaviors that serve to minimize free energy (Ramstead et al., 2020). In short, the generative model couples an agent’s internal states, (which *embody the system’s internal dynamics*), with the external states in which the agent is situated or embedded (Ramstead et al., 2020).

Mathematically, belief updating (moving from prior beliefs to “posterior” beliefs) involves an inversion of the likelihood element of the generative model, (from p(o*│*s), to p(s*│*o), i.e., mapping from consequences to causes) under prior beliefs, p(s). If we normalize the above equation by dividing the generative model by the overall probability of observations—a.k.a., model evidence (or marginal likelihood)—p(o), we then have Bayes’ theorem:

$$p(s│o)=\frac{p(o\;\;\;│s)\cdot p(s)}{p(o)}$$

In plain language, Bayes theorem here describes inference about states of the world given some observations, p(s*│*o), as being proportional to the likelihood of some observation given a particular state times the prior probability of that state. This describes Bayesian inference, or the process by which percepts (posterior beliefs) are updated from prior beliefs—given some observation or sensory evidence—under a generative model. Heuristically, Bayesian belief updating is the changing of one’s perceptual experience to best explain sensory observations. In predictive coding formulations, this can be construed as using precision-weighted prediction errors to update posterior beliefs that resolve or explain away prediction errors.

Active inference takes this a step further by incorporating an agent’s actions that serve to minimize the discrepancy between predictions and sensory outcomes; not solely by changing predictions but also by acting to change sensory samples (Smith et al., 2022a). This discrepancy is scored by (variational) free energy or precision-weighted prediction error (Smith et al., 2022a). In other words, active inference provides an account by which an agent, with embodied beliefs, engages in active sense-making to resolve uncertainty (minimize variational free energy) under a generative model that serves to guide action, which in turn, updates perception (beliefs). Thus, the embodied agent makes sense of the world through perception-action cycles guided by prior beliefs. This is not intended to be a detailed or formal computational account but rather to motivate a relatively straightforward formal framework that supports analytic and numerical studies.

The introduction of Bayes’ theorem allows us to consider belief updating as a basis for building the active inference story. Briefly, with mathematical treatment and (mild) theoretical assumptions, we can posit basic, qualitative active inference equations, based on Bayes’ theorem, that cast perception and action as minimizing variational and expected free energy, respectively. In this setting, one can read the respective free energies as statistical constructs, where (Parr et al., 2022):

Variational Free Energy=Complexity+Inaccuracy

Expected Free Energy=Risk+Ambiguity

Perception—described as minimizing variational free energy—affords the most accurate and parsimonious explanation for sensory observations; namely, those that resolve precise prediction errors (i.e., increasing accuracy) as simply (i.e., decreasing complexity) as possible. In Bayesian terms, complexity is how much updating the prior undergoes to update beliefs, while accuracy is the expected (log) likelihood of the observation. Action plans (i.e., policies), read as minimizing expected free energy, avoid surprising outcomes in the future—by minimizing risk (expected complexity)—and resolving uncertainty (i.e., expected inaccuracy, or surprise) by minimizing ambiguity. For a tutorial on the mathematical and theoretical development of these concepts, the reader is referred to Smith et al. (2022a).

For the purposes of our clinically oriented discussion, we assume that active inference provides a formal mathematical and theoretical account of how an agent, through action and perception cycles, maintains an optimal and adaptive fit between itself and the environment; i.e., realizing prior beliefs about the kind of thing it is—within its given environment—through (Bayesian) modeling, predicting, evaluating, and acting.

We will now shift our attention to the clinical implications of the “active ingredients” of active inference, attempting to integrate the lived experience of the person with dizziness into the discussion. For our purposes, we will read the minimization of free energy as the minimization of precision-weighted prediction errors, noting that the requisite precisions also have to be predicted under a person’s generative model.

### Clinical significance of the generative model

In active inference, the generative model is essentially a specification of an agent’s prior beliefs about the states of the world and how they cause sensations (Smith et al., 2022b). In order to have effective agency—or the ability to control one’s actions in their environment (Haggard, 2017)—the organism, here a person with dizziness, must implicitly predict (model) the consequences of their actions based upon prior information or experience (Ciaunica et al., 2022). Using priors to predict the consequences of actions can be read as planning (Kaplan and Friston, 2018). The more detailed and accurate the model (and its predictions), the more effective the agentive behavior (Kaplan and Friston, 2018). This is consistent with the premise of the good regulator theorem (Conant and Ross Ashby, 1970; Friston, 2010; Friston et al., 2010), which states that “every good regulator of a system must be a model of that system.” Therefore, a good generative model, that is, one that serves as a good control system, will, over time, *attune* the agent to its environment (Ramstead et al., 2020). In terms of enactivism and ecological psychology, a good generative model includes affordances that couple intention—i.e., prior beliefs about the consequences of action—and precision within the environment (Luu et al., 2024).

Clinically and phenomenologically, the generative model of a person with persistent dizziness may reflect a state of uncertainty; owing to its inability to accurately predict outcomes with precision. Through hierarchical message passing, the ensuing uncertainty propagates across multiple domains, including perception, action, agency, and emotional affect, thereby offering a vantage point through which the complexity and breadth of a person’s conscious (lived) experience can be observed, appreciated, and perhaps understood.

## Active inference framework of persistent dizziness

### Sensory attenuation

In predictive coding formulations of active inference, since prediction errors—ascending inputs unexplained by descending (generative model) predictions—drive perception, they are essentially the newsworthy part of sensory signals (Seth and Friston, 2016). The agent’s internal dynamics serve to minimize prediction errors (or maximize model evidence) in a manner consistent with the policies set forth under prior beliefs entailed by the generative model. Prediction errors can be minimized through perception, in which the generative model is updated, or by action, whereby predictions are fulfilled (Friston, 2010; Friston et al., 2010). In order to realize an intended act, sensory evidence (i.e., prediction errors) indicating one is not acting must be attenuated. Thus, action depends on sensory attenuation, which rests on attenuating the precision of sensory prediction errors (Adams et al., 2013). Sensory attenuation is understood as a necessary mechanism for normal action and perception (Limanowski et al., 2024; Limanowski and Friston, 2020a, 2020b), and moreover for one’s sense of agency: as sensory attenuation is necessary for attribution of actions as being self-generated (Ciaunica et al., 2022). A well-described example of sensory attenuation is saccadic suppression—the transient suspension of visual input, such that we do not perceive the optic flow during the self-made act of moving our eyes (Wurtz, 2008; Crevecoeur and Kording, 2017). There are many other examples of sensory attenuation, ranging from attenuation of self-produced vocalizations (Friston and Frith, 2015) to vestibular suppression during self-generated head movements, including gaze shifts (Roy and Cullen, 1998; Cullen et al., 2004; Cullen, 2023), in which saccadic eye movements occur in the same direction as head movements, necessitating the attenuation of sensory afferents associated with vestibulo-ocular reflex (VOR): if unattenuated, eye movements contralateral to head movements would oppose the intended gaze shift (Roy and Cullen, 1998; Cullen et al., 2004; Cullen, 2023). During these self-generated head movements, vestibulospinal sensory afferents must also be attenuated, otherwise the intended movement would be opposed, and the person would experience conflicting self-motion perceptual cues (Cullen, 2023; Cullen, 2019), i.e., evidence that they are not moving. Taken together, these examples of sensory attenuation point to the crucial role sensory attenuation plays in action and perception across multiple systems.

### Sensory precision weighting / failure of attenuation as sustaining mechanism

In the context of acute dizziness, for example vestibular neuritis, in which there is sudden, unilateral disruption to the balance of bilateral vestibular inputs, unpredicted vestibular inputs (i.e., prediction errors) are likely to update beliefs about perceptual stability, generating new predictions that reflect low confidence (i.e., a loss of precision) about balance. Sustained uncertainty—imprecise priors—can lead to overly precise sensory (proprioceptive, visual, vestibular) prediction errors, manifesting as excess attention to bodily sensations (Staab, 2023; Arshad et al., 2022; Edwards et al., 2012). Crucially, imprecise priors, or predictions of excess precision can occur in the presence of normal peripheral inputs. Excessive precision weighting of sensory prediction errors, occurring solely in the context of uncertain priors—e.g., when under stress—(Peters et al., 2017), may result in a failure of sensory attenuation, resulting in dizziness, imbalance, and altered gaze control, all consistent with the pathophysiology of persistent dizziness (Staab, 2023).

Patients who experience persistent dizziness show evidence of excessive precision weighting of sensory prediction errors, through sustained decreases in sensory thresholds (Wurthmann et al., 2021). In other words, regardless of initial cause, persistent dizziness may be maintained by a failure of sensory attenuation. There are many conditions that yield to an explanation in terms of a failure of sensory attenuation: e.g., (Rossi et al., 2025; Blakemore et al., 1999; Brown et al., 2013; Kube et al., 2020; Limanowski, 2017; Edwards et al., 2012; Pareés et al., 2014). Mechanistically, active inference thus casts persistent dizziness as a kind of *false inference that occurs in the context of excessive sensory precision.* Put simply, people experiencing persistent dizziness have lost the capacity to ignore vestibular prediction errors that would normally be attenuated; especially, when induced through movement. These unattenuated prediction errors must be explained, resulting in the perceptual hypothesis (updated posterior belief) “I am dizzy”.

## Expectation violation: lessons from altered sensory environments

Our account connects with the clinical-psychological literature on expectations. The Violated Expectations (ViolEx) model describes updating violated expectations (accommodation), discounting disconfirming experience (immunization), and acting to confirm expectations or avoid further violation (assimilation and anticipatory behavior; Rief et al., 2015; Panitz et al., 2021). In active inference terms, these processes concern prior beliefs, prediction errors, belief updating and action selection. Expectations such as ‘moving my head will make me dizzy’ can be held with considerable confidence, while their revision depends on the weight afforded to new experience. Expectation violations are closely related to prediction errors (D'Astolfo and Rief, 2017), and Kube et al. (2020) link immunization in persistent physical symptoms to overly precise priors that override benign bodily evidence. Expectations and avoidance have been implicated in symptom maintenance (Löwe et al., 2024), with related accounts in musculoskeletal pain (Rossettini et al., 2022) and expectation violation proposed as a treatment mechanism (Rief et al., 2022).

This emphasis on attenuated evidence may appear inconsistent with our proposal of failed sensory attenuation. We suggest that the accounts are complementary: evidence challenging symptom expectations is discounted, while self-generated sensory prediction errors that appear to confirm them remain unattenuated. Both privilege evidence of threat. Uncertainty about how movement will feel can therefore coexist with confidence that it will provoke dizziness: ‘predictable unpredictability’.

Altered game environments provide a concrete example. Experienced players exposed to rotated or mirrored environments reported headache, nausea, dizziness and feelings of unreality, sometimes within seconds; laboratory studies involving passive viewing also reproduced symptoms (Kokkinakis et al., 2025). The authors termed this ‘expectancy violation headache’ (EVH). Within our framework, extensive practice establishes precise expectations about the environment. Familiar features, when experienced in unexpected spatial relationships may generate sustained precision-weighted prediction error and familiar actions may no longer resolve the mismatch. Less experienced players appear less affected, consistent with evidence that expertise can amplify uncanniness (Diel and Lewis, 2022) or other experiences, including headache (Singh, 2018, as discussed by Kokkinakis et al., 2025). Feelings of unreality also resonate with accounts linking presence to predictions of self-related signals (Seth et al., 2012) and active inference approaches to depersonalisation (Ciaunica et al., 2022).

Related phenomena occur with inverted or reversed vision, which can initially provoke disorientation, nausea and unreality before adaptation (Stratton, 1896, 1897; Dolezal, 1982, as discussed by Bredlau, 2011; Degenaar, 2014). Optical reversal can retune or reverse the vestibulo-ocular reflex (Gonshor and Melvill Jones, 1976), while the contribution of self-produced movement to visual adaptation supports our emphasis on updating through action (Held and Freedman, 1963). Aniseikonia is associated with headache, dizziness and fatigue (Burian, 1943). Virtual reality can increase depersonalisation and derealisation (Aardema et al., 2010; Peckmann et al., 2022), experiences also more frequent in vestibular disease (Yen Pik Sang et al., 2006; see Cento et al., 2026).

Adaptation may itself make ordinary surroundings unexpected, as illustrated by prism after-effects (Held and Freedman, 1963) and the broken escalator phenomenon (Bronstein et al., 2009). In Mal de débarquement syndrome, oscillatory sensations emerge after passive motion ends and temporarily ease when motion resumes (Cha et al., 2020), consistent with continued prediction of the adapted motion. In EVH, the mismatch has an identifiable external source, and leaving the game or returning to the familiar map can end exposure. In our account of persistent dizziness, it accompanies movement and may be attributed to the body. Avoidance, stiffened posture and reliance on support may limit opportunities for updating. Successful movement may then be dismissed as a good day or credited to holding on, preserving the expectation of dizziness rather than restoring confidence (Kube et al., 2020; Panitz et al., 2021).

Whether expectations change also depends on their certainty, attention, emotion and the credibility of new information (Rief et al., 2015; Panitz et al., 2021). Within our framework, these factors influence the precision afforded to prior beliefs and new evidence, helping explain why anxiety, vigilance and trust in the clinician may matter. Some players nevertheless found transformed environments enjoyable during exploration (Kokkinakis et al., 2025). This accords with proposals linking affective valence to confidence in resolving uncertainty through action (Joffily and Coricelli, 2013; Hesp et al., 2021). Chosen, bounded uncertainty may therefore feel different from uncertainty that is imposed and appears irresolvable.

Players’ accounts of sickness with on-screen movement and apparent falling (Kokkinakis et al., 2025) resonate with the seasick-like instability described in our clinical vignette. Familiar environments felt dream-like or strangely unfamiliar, recalling the uncanniness discussed above. Depersonalization and derealization are also more frequent in vestibular disease, although this evidence is not specific to persistent dizziness (Yen Pik Sang et al., 2006). Players’ repeated attempts to mentally restore the scene, difficulty with simple tasks and tiredness offer further parallels with the effortful control and exhaustion described by people with persistent dizziness (Gamble et al., 2023).

These similarities concern lived experience and do not establish a shared mechanism. The players were healthy, their symptoms were transient and largely visually provoked, and laboratory participants watched rather than played. Nevertheless, the comparison helps distinguish an identifiable environmental mismatch from one experienced as arising within the moving body. We propose that the latter may be harder to resolve when avoidance limits disconfirming experience and successful movement is discounted rather than used to update expectations (Kube et al., 2020; Panitz et al., 2021).

## Active inference and the lived experience of the person with persistent dizziness

### Precision and uncertainty

Interpreted through an active inference lens, the lived experience of a person with persistent dizziness (disrupted agency, invalidation, difficulties interpreting symptoms, uncertainty about the future, emotional distress, and a persistent struggle for control and coherence (Gamble et al., 2023; Sezier et al., 2019; Olsson Möller et al., 2014; Kruschinski et al., 2010; Mendel et al., 1997) can be seen as a phenomenon in which generative models of the self, environment, and future have settled into an “attractor state” of irreducible uncertainty—a state of predictable unpredictability. This state is maintained by the excessive precision (i.e., attention) afforded to prediction errors across various domains (Edwards et al., 2012); either due to a failure of sensory attenuation, or as a compensatory increase in the precision of prediction errors.

Psychologically speaking, this pernicious failure of sensory attenuation is consistent with explanations of “hypervigilance,” in which the person is unable to ignore their sensorium (Owens et al., 2018; Friston, 2023), meaning that evidence for an uncertain world is regarded as the “new normal.” Importantly, this formulation unifies previously dichotomized “vestibular symptoms” and “psychological distress,” by framing both as reflections of the extent to which uncertainty remains unresolved by perceptual and deep interoceptive generative models, respectively. Prediction errors that are persistently unattenuated—i.e., unresolved—can manifest as a persistent state of uncertainty (anxiety; McGovern et al., 2022) and confound one’s ability to regulate internal states across multiple domains (allostatic self-efficacy): inducing beliefs which are consistent with depression (Stephan et al., 2016).

While it is reasonable to assume that premorbid “priors” of anxiety and depression affect one’s beliefs about recovery from acute dizziness, we contend that emotions are phenomenologically intrinsic to the experience of persistent dizziness and are therefore not mechanistic, isolated targets for intervention. Evidence for use of selective serotonin reuptake inhibitors (SSRIs) and serotonin-norepinephrine reuptake inhibitors (SNRI’s) for the treatment of persistent dizziness is lacking (Webster et al., 2023). Moreover, benzodiazepines were found to be no more effective than antihistamines or placebo in treating acute vertigo (Pettit et al., 2023).

Hesp et al. (2021) provide a formal computational account of the interconnections between affect, perception, and planning, under active inference. In this account, anxiety reflects uncertainty, read specifically as low confidence in the ability of one’s action model (i.e., policies) to minimize expected free energy (i.e., divergence between preferred and expected outcomes). Low confidence in action—as a way of reducing ambiguity (uncertainty)—results in inaction, thereby (by default) increasing the influence of prior beliefs that uncertainty cannot be resolved. In the context of uncertainty, characterized by imprecise priors (and therefore predictions), sensory prediction errors may be afforded excessive precision, and thus are unlikely to be attenuated. This evinces a vicious circle, in which one is attending to evidence that the sensorium is unpredictable (high risk)—and that uncertainty cannot be resolved actively (c.f., learned helplessness). Importantly, if one’s priors include negative affect (anxiety and / or depression), then a self-maintaining cycle of inaction and unresolved uncertainty is more likely to ensue. Active inference models have also been linked to psychological theories of fear avoidance. These models suggest a circular causality: fear is inferred from physiological and contextual cues, and this fear hypothesis then drives avoidance behaviors (inaction), which in turn reinforce the original fear and uncertainty (Allen et al., 2022). This circularity aligns with the idea that low confidence in action (due to fear or negative affect) leads to avoidance, which maintains or increases uncertainty and negative priors (Allen et al., 2022).

Under the active inference formulation, prior beliefs reflect baseline ambiguity (uncertainty). Previously, we discussed prior beliefs as a pre-onset baseline prediction of the generative model. While premorbid priors are informative, from a treatment standpoint, we consider prior beliefs as those held (consciously by the patient) prior to treatment (i.e., after the onset of symptoms). When novel sensory inputs (i.e., prediction errors) associated with dizziness episodes are encountered, a premorbid prior belief is revised into a posterior belief, thereby minimizing prediction error by updating the generative model, which propagates forward a new (empirical) prior, before the initiation of treatment.

Drawing from lived experience data, a person with dizziness may express prior beliefs reflecting disrupted agency, for example, “I feel as if I am not in control over my balance. The ground feels as if it’s moving beneath me.” Not all beliefs are consciously held by a person, nor do they necessarily manifest in a “top-down” manner. At the subpersonal level, prior beliefs (predictions of sensations or intent) may encounter unexpected, imprecise, or unattenuated sensory inputs (prediction errors) or be unfulfilled by actions, leading to an altered sense of self and, therefore, agency (Ciaunica et al., 2022; Pareés et al., 2014). Thus, active inference provides a hierarchical bidirectional framework for message passing that ultimately connects subpersonal beliefs with intentional behaviors (Friston, 2023).

Crucially, failed sensory attenuation (excessive weighting of prediction errors related to visual, vestibular, proprioceptive, or integrated prediction errors) is not a unidirectional phenomenon, regardless of the original source (i.e., peripheral or central). Indeed, lower vestibular perceptual levels have been identified in people with persistent dizziness (PPPD) with normal peripheral vestibular function (Wurthmann et al., 2021), suggesting failed sensory attenuation without a clear directional instigator. Due to circular causality, once established, precision weighting is *both* top-down (prediction driven) *and* bottom-up; that is predictions manifest prediction errors, which in turn, manifest new predictions (Friston et al., 2016). Mathematically and pragmatically the result is identical; prediction errors are given greater precision weighting, changing thresholds that control peripheral reflexes, perpetuating unresolved uncertainty resulting in (and from) belief updating that supports continued excessive precision weighting (Friston et al., 2016), manifesting as self-evidencing beliefs reflected in lived experience: “…I have to stay in my safe place.” (Gamble et al., 2023).

Importantly, this may serve to reinforce avoidance behaviors that preclude foraging for the kind of sensory evidence needed for updating these beliefs. In a behavioral model of uncertainty (anxiety), optimal agents exhibit exploratory actions—increased information seeking—in attempts to resolve uncertainty (Hesp et al., 2021). Properly guided, action-oriented exploratory approaches may be pro-adaptive, in direct contrast to avoidance behaviors common to conditions such as persistent dizziness.

## Discussion: active inference-informed treatment

### Therapeutic interactions

In this perspective, we have focused on the holistic aspects of the active inference formulation of persistent dizziness. Active inference models rest on hierarchical generative models, with message passing of increasing depth (from subconscious to conscious) and breadth (from posture to emotions) of sense-making (Pezzulo et al., 2024b). This highlights the different ways in which such formulations can be deployed therapeutically. Generally speaking, through shared (extended cognitive) interactions between the patient and provider, these formulations can be used to facilitate an (en)active and embodied sense-making process, consisting of exploration of movement, attentional strategies, and belief updating in the various contexts in which they are embedded. Tending to a person’s beliefs about agency through validation and therapeutic alliance, their beliefs about abilities (self-efficacy) through successful skill acquisition, and their beliefs about precision through attentional focus strategies are among the many value applications of this unifying perspective that need to be explored. This reframing establishes the pre-eminent role of the clinician as a facilitator of successful active sense-making (enactive belief-updating) experiences in their clinical engagement with patients living with persistent dizziness. While active inference consists of iterative perception-action loops that are ostensibly continuous, we will explore and elaborate on a logical implementation sequence that one might deploy in the clinic.

### Restoring agency

*Agency*, or the feeling of being in *control* of one’s bodily actions (Haggard, 2017), is foundational within an active inference framework and is therefore a key therapeutic target. First-person (lived experience) accounts of persistent dizziness reveal disrupted agency (Gamble et al., 2023; Sezier et al., 2019; Olsson Möller et al., 2014; Kruschinski et al., 2010; Mendel et al., 1997). A clinically logical starting point for restoring agency is the establishment of a productive therapeutic alliance (McParlin et al., 2022; Stilwell and Harman, 2021). Through an active inference account of the therapeutic alliance, Kim et al. (2022) describe how *validation* occurs through the development of a shared narrative (a revised generative model) between the provider and patient, thereby addressing the expressed lived experience problem of invalidation—often as a result of interactions with health care providers—as described by phenomenological research (Stilwell and Harman, 2021; Gamble et al., 2023; Sezier et al., 2019; Olsson Möller et al., 2014; Kruschinski et al., 2010; Mendel et al., 1997). A therapeutic alliance that features provider empathy and communication of positive outcome expectations has been shown to improve outcomes for a variety of conditions (Howick et al., 2018). Experiential coupling to the self and environment through therapeutic touch (Kim et al., 2022), physical exercise, and manual tasks (Ciaunica et al., 2022) are suggested strategies for restoring agency from an active inference perspective. At the intersection of task performance and agency, motor learning paradigms (i.e., OPTIMAL)^5^ in which an agent can choose their own tasks and manner of engagement (autonomy support) have been shown to increase their self-efficacy beliefs and associated outcomes (Wulf and Lewthwaite, 2016).

### Belief updating

While much (if not most) of the overall (Bayesian) belief dynamics that sustain persistent dizziness are not consciously manifested, therapeutic interactions and action plans may effectively engage with a person’s conscious beliefs and associated behaviors. From an active inference standpoint, the mechanics of belief updating are governed by the same process, regardless of whether these beliefs are subpersonal or conscious (Friston, 2023). There are many candidates for the types of consciously held beliefs that are important to address clinically, including illness beliefs, self-referential beliefs, and perceptions of ability. Among all the potential options for addressing conscious beliefs, expected outcomes, or recovery beliefs, have been shown to be the most robust predictors of recovery from persistent dizziness (Katzenberger et al., 2023; Herdman et al., 2022). Specifically, self-efficacy, or the belief that one can deploy and sustain the behaviors necessary to produce the desired outcome (Bandura, 1977), has been shown to predict positive outcomes for dizziness (Katzenberger et al., 2023). Moreover, self-efficacy beliefs are ubiquitous predictors of outcomes in various health-related challenges, such as pain (Costa et al., 2011), falls (Landers et al., 2016), stroke (Korpershoek et al., 2011), and frailty (Hladek et al., 2021). Crucially, self-efficacy is improved through mastery experience (Bandura, 1977), thus casting the role of intervention as a successful sense-making process in which a person with persistent dizziness engages in exploratory (i.e., ambiguity minimizing) movement behavior, preferably involving valued activities designed to increase perceptions of success (i.e., risk minimizing). The intervention is reframed by active inference as a process of resolving uncertainty, read as minimization of expected free energy through actions that maximize exploration. An example of this process is included in the clinical vignette (Box 1).

BOX 1 **Clinical vignette.**

“John,” a 69 year old man, describes his 20 + year experience living with persistent dizziness.

Themes that emerge from the patient’s narrative account:

- Interpreting symptoms in light of their effect on identity (self-evidencing).
- Failed sensory attenuation.
- Avoidance behavior (to minimize risk and ambiguity).
- Action as a means for (gradual and partial) sensory attenuation.
  - Valued (preferred, risk-minimizing) activities.
- Belief updating though action – beliefs about abilities, outcomes, identity.

MB: What were the symptoms like when they first came on?

*Well…I’m a woodworker….so I do a lot of work in my wood shop, and so I’d be working at doing a task, and I would start to feel not very well balanced or a little woozy…kind of like, I think, what people experience when they get seasick. You know, like the ground is sort of moving below you. You’re kind of swaying. And that was unsettling, but it was brief, and I could then continue with my work and sort of very happily ignore the fact that something unpleasant had just occurred… until it got to the point where it became more persistent and I had to recognize what was going on…I began to get really tired and then I would need to rest. When I would just lay down on a bed, the symptoms would abate, and I could just lie there quietly and happily relieved of the symptoms - but sometimes I would take a three-hour nap, and then as soon as I went from lying down to sitting up, the symptoms would come right back and they’d be just as bad. And that was very depressing…I started to become depressed about it and think this is the way my life is going to be from here on out. There’s no way, there’s no way out of this. And that was hard.”*

Therapy addressed the feeling of imbalance with valued (risk minimizing) activities involving movements that he avoided because they produce symptoms; importantly movements he wanted to return to (e.g., yoga and balance exercises).

*“At first, I was…not really paying attention to what was happening. It wasn’t pleasant because it was exacerbating the motion sickness, the dizziness that I felt. But I also trusted that what we were doing made sense, despite the fact that it was actually making the condition worse. And then, as I got more* into *it…my condition started to improve…*

*…I feel like that’s since that, I’ve felt like much more capable of handling it. And when it does happen, instead of avoiding it, which was, as I mentioned, the way I did in the past, I do things to intentionally meet it….like yoga poses, balancing exercises…movements that challenge the feeling of dizziness, of motion sickness…instead of doing that before I would just lie down to relieve myself of the symptom. And when I do that, I can deal with it better. It doesn’t eliminate the symptoms, but it kind of makes me more comfortable with the fact that they exist and that at least I’m doing something positive to try to deal with the problem. And I think that even if the problem doesn’t go away, it almost immediately gets better, and I feel much better about it. I feel more optimistic that there’s a solution or at least I can control it and live with it comfortably because I’m not living in fear. I’m actually, you know, actually happy again and optimistic that I can live my life with this symptom and live it happily and do the things that I like to do*.

In ViolEx terms, these interventions may support updating when disconfirming experiences are credible and attributed to the person’s own capacities, rather than discounted or credited to safety behaviors (Rief et al., 2022). Inhibitory learning approaches to exposure similarly emphasize testing feared outcomes rather than relying on habituation alone (Craske et al., 2014). A valued but symptom-provoking movement can therefore become a behavioral experiment: for example, testing the expectation that turning one’s head while walking through a supermarket will lead to a fall. Reviewing the outcome helps establish what was learned and whether success was dismissed as a good day or attributed to holding the trolley. The therapeutic relationship also matters, as perceived clinician warmth and competence can support the revision of negative expectations (Seewald and Rief, 2023).

Our clinical vignette can be understood in these terms. The patient initially saw no way out of his symptoms and managed them by lying down. Through valued movements that initially provoked symptoms, he came to approach experiences he had previously avoided. This suggests updating at the level of agency and self-efficacy: greater confidence in coping and renewed optimism, even while symptoms remained. Some players’ enjoyment of transformed game environments (Kokkinakis et al., 2025) also raises the possibility that framing graded exposure as self-directed exploration may change how uncertainty impacts the individual. This interpretation accords with OPTIMAL theory’s emphasis on autonomy and enhanced expectancies (Wulf and Lewthwaite, 2016).

### Expected outcomes (likelihood)

A person’s ability to plan and execute intentional actions that afford informative and characteristic (preferred or goal-directed) behavior, depends upon the accuracy of their generative model’s predictions of the *likely* sensory consequences of a given action plan or policy. If one feels dizzy most of the time, or their symptoms have generalized triggers, they may conclude that *any* action is a potential cause of their symptoms and have low confidence that their generative model can bring about a preferred outcome. The person may reinforce this conclusion through action avoidance, thereby sustaining the deprivation of the potential to actively generate preferred observations or evidence that they are no longer attainable. Thus, illness behaviors and associated experiences are learned. Realizing preferred outcomes under a given action plan (policy)—where preferred outcomes can themselves be revised, i.e., preference learning (Da Costa et al., 2025; Sajid et al., 2022)—is enacted through successful movement experiences in which preferred (unsurprising) observations are solicited through engagement in valued tasks with high epistemic value to the patient, while leveraging attentional strategies that affect precision weighting mechanisms that can plausibly alter the symptomatic experience; thereby eluding surprising, uncharacteristic or unpreferred observations.

### Precision (attention): recovery mechanism and therapeutic target

Recovery from acute peripheral vestibular syndrome (i.e., vestibular neuritis) is understood as initiated by reductions in contra-lesional peripheral (vestibulo-ocular) reflex responses (decreased synaptic gain), thereby restoring perceptual symmetry (Cousins et al., 2013). In active inference terms, this is construed as the minimization (attenuation) of sensory prediction errors through precision weighting. This illustrates the crucial role sensory attenuation plays in both recovery and persistence (via its failure), making it a potentially productive target for therapeutic intervention.

### Attentional strategies

The precision weighting mechanism is understood as synaptic gain, with attention being the process by which this gain manifests (Feldman and Friston, 2010). Thus, attentional strategies may provide opportunities for patients to address their symptoms more effectively (Herdman et al., 2022). One approach could be construed as enabling patients to mentalize their precision control in terms of attentional selection (e.g., Duquette, 2020; Fotopoulou and Tsakiris, 2017); in other words, develop volitional strategies to skillfully attend to or attenuate the sources of evidence for their experienced states of being: c.f., (Limanowski, 2017; Limanowski, 2022).

Focusing one’s attention externally has been shown to have positive effects on motor performance (Wulf and Lewthwaite, 2016), including balance (Kim et al., 2017; Chiviacowsky et al., 2010; Kee et al., 2012), perceptions of stability (Ellmers et al., 2021), sensory conflict resolution (Ma et al., 2022), and postural control strategies in the context of persistent dizziness (Wuehr et al., 2017). Moreover, task-directed (externally focused) attentional strategies have been shown to decrease precision weighting of visual inputs relative to proprioceptive inputs (Limanowski and Friston, 2020a, 2020b; Ma et al., 2022), which is promising for individuals with persistent dizziness, who commonly experience increased visual sensitivity due to excessive precision afforded to sensory inputs in the context of low confidence in vestibular inputs, referred to as “visual dependence” (Staab, 2013; Dieterich and Staab, 2017; Staab, 2023). Thus, a task-directed (external) attentional focus is itself a strategy for sensory attenuation and for regaining appropriate precision control of bodily movements (Limanowski, 2017; Zachry et al., 2005). There is evidence that attentive absorption into a highly skilled, well-rehearsed task underpins the attainment of “flow state” wherein a person experiences “wholesale” sensory attenuation by attending to sources of sensory information outside the self (Parvizi-Wayne et al., 2024).

Another approach involves the engagement of subpersonal mechanisms of sensory attenuation, to gain indirect volitional control over the precision of vestibular prediction errors. The rationale here rests upon the role of sensory attenuation in permitting volitional execution of intended acts: to realize a prior intention, it is necessary to suspend the precision of the vestibular, proprioceptive, and exteroceptive consequences of the intended act (Cullen, 2023; Kilteni et al., 2025; Limanowski et al., 2024). These sensory modalities are united under a generative model capable of predicting and therefore attenuating the precision of prediction errors associated with the multisensory consequences of a particular action policy. This is of potential therapeutic value for the person with dizziness, given emerging evidence that there is a heightened multimodal sensory sensitivity in effect (Powell et al., 2020). Because sensory attenuation covers all sensory consequences of action, it can be used to attenuate modalities that are not under volitional control. For example, rubbing your tummy when you have a stomach-ache would be an example of inducing sensory attenuation on interoceptive and nociceptive inputs, in conjunction with the proprioceptive attenuation induced by voluntary limb movement. This example is suggestive in the sense that some therapeutic interventions for persistent dizziness can be framed in terms of volitional actions (e.g., adopting a particular posture) that would, in principle, induce sensory attenuation of the vestibular and proprioceptive prediction errors mediating the felt dizziness. Specifically, vestibulospinal afferent attenuation occurs during walking, with greater attenuation occurring at increased walking speeds (Dakin et al., 2013). This has implications for attenuating dizziness symptoms, as vestibulospinal afferents transmit sensory observations used in self-motion perception (Cullen, 2023; Cullen, 2019).

Taken together, treatment approaches that involve active, meaningful-task-focused strategies appear highly compatible with an active inference framing. This calls upon the previously discussed concept of affordances, in which the person engages their embedding environment in accordance with its action possibilities, thereby maximizing agentive behavior and congruence between self, environment, perception, and action.

### Action

According to Bandura (1977), mastery experiences facilitate positive self-efficacy beliefs. This places the provider in the role of promoting (and defining) successful actions undertaken by the person with persistent dizziness. As discussed, actions are predicated on sensory attenuation (Blakemore et al., 1999; Cullen et al., 2004; Adams et al., 2013; Limanowski, 2017; Cullen, 2023), including sensory inputs related to self-motion (Cullen et al., 2004; Cullen, 2023). This supports the use of action-based recovery strategies. Moreover, belief updating is an enacted process, predicated on actions that bring about preferred observations. A related example of belief updating as being enacted is the “broken escalator phenomenon”, in which the same motor strategy that occurs when stepping onto a moving escalator, known as the locomotor aftereffect (truncal overshoot), continues to be enacted despite conscious awareness of the escalator belt being shut off (Bronstein et al., 2009). Interestingly, this aftereffect, also a kind of false inference, was reduced when participants engaged in an attentional task while being exposed to the moving escalator (Patel et al., 2014).

Active inference adds a depth of modeling that appeals to the scale-invariant and interconnected nature of subpersonal beliefs about action policies (and associated movement trajectories) and consciously held beliefs about the abilities of these policies to bring about a desired outcome (Friston, 2016a; Friston, 2016b). It also resolves the biological plausibility problem of previous models in that—unlike cost functions in optimal motor control models, which are computationally intractable in real time—prior beliefs emerge naturally from generative models controlling sensorimotor loops, forming a readily deployable action plan (Friston, 2011). Optimizing beliefs about one’s ability to achieve preferred outcomes, the result of enacting an effective generative model capable of minimizing expected free energy, aligns with the concept of high self-efficacy beliefs, positive emotional valence, and subjective well-being (Smith et al., 2022b). Thus, when optimized, active inference *is* the process of achieving preferred outcomes through successful agentive and active engagement of the person’s lived body within their lived world, thereby improving their lived experience. An active inference framing of an exemplary patient’s lived experience of dizziness and therapeutic intervention is provided in the clinical vignette (Box 1).

### Illustration

See Figure 1.

**Figure 1:**
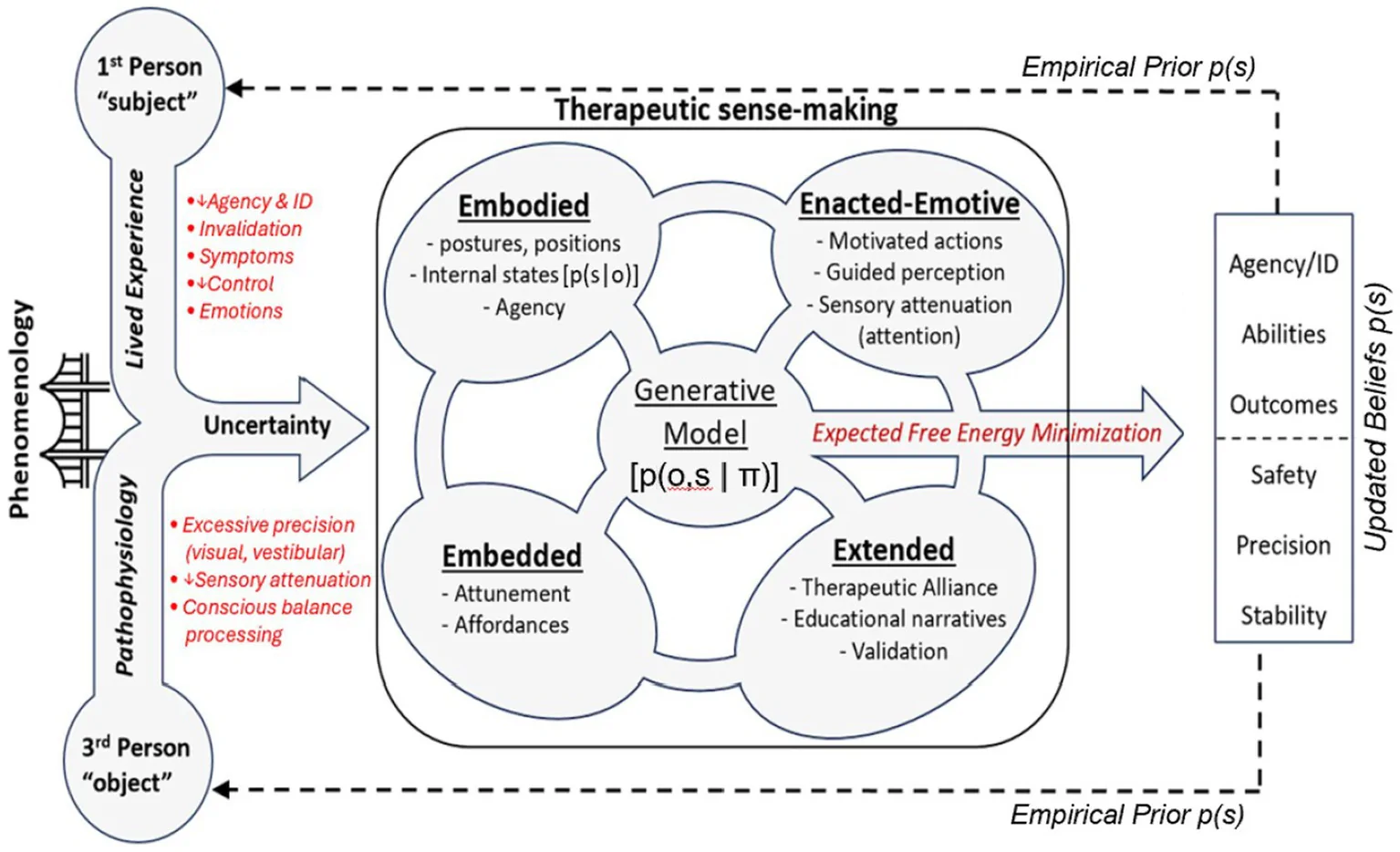
Phenomenology serves as a bridge between first and third person perspectives. A unified theme of “uncertainty” emerges under active inference. The center reflects the 4E sense-making process informed by enactivism and ecological psychology by which uncertainty is resolved (free energy is minimized) through patient-provider interaction. This results in updated beliefs influencing both the objective process and lived experience.

### Compatibility of active inference with contemporary motor learning theory: OPTIMAL

The “active ingredients” of active inference—agency, beliefs, and precision—align well with OPTIMAL (optimizing performance through intrinsic motivation and attention for learning) theory (Wulf and Lewthwaite, 2016). The OPTIMAL theory provides a clinically accessible framework through which autonomy and expectations (“motivated beliefs”) are supported and enhanced, respectively, while attention is externalized to the motor task (Wulf and Lewthwaite, 2016). When these three factors are ‘optimized’ simultaneously, the effects on motor performance are additive (Wulf et al., 2017). OPTIMAL theory has been applied to clinical populations, such as those with MS (Khalaji et al., 2025), stroke (Sánchez and Winstein, 2021), and sports injury rehabilitation (Gokeler et al., 2023).

## Conclusion

An active inference account of persistent dizziness offers an integrative framework that bridges enactivist and ecological psychology models with the observable phenomena of the condition, including first-person accounts from those who experience it. By anchoring the lived experience of persistent dizziness, which is characterized by disrupted agency, invalidation, difficulties in interpreting symptoms, uncertainty, emotional distress, and a persistent struggle for control and coherence within a principled theoretical account, this framework supports a more holistic, person-centered approach to clinical understanding and engagement. Importantly, this account does not oppose existing treatment strategies; rather, it provides an overarching conceptual scaffolding within which established approaches, such as vestibular rehabilitation, cognitive behavioral therapy, and psychoeducation, can be situated, understood, and potentially extended. This type of theoretical synthesis, drawing on active inference, enactivism, and ecological psychology to organize clinical practice around a unified account of the person-environment relationship, has demonstrated conceptual traction in adjacent healthcare disciplines, lending further plausibility to its application here. This account was motivated by our recognition that existing models of persistent dizziness, while increasingly biopsychosocial in orientation, have yet to fully incorporate the phenomenological dimension of the condition into a theoretically coherent whole. Our primary aim was, therefore, to present this formulation as an attempt to illuminate the treatment strategies most consistent with an active inference perspective and to make that account accessible to clinicians working with this population. We acknowledge that the framework presented here is hypothetical in nature, and that we have not proposed specific empirical methodology to evaluate its claims. This is a limitation we share with foundational or preparatory theoretical contributions in this space, and one we regard as a necessary first step rather than a final destination.

The expectancy-violation paradigm of Kokkinakis et al. (2025) offers a starting point for testing our account. We predict that people with persistent dizziness will show stronger and more slowly resolving responses to transformed familiar environments, such as mirrored footage of their home or neighborhood, than matched controls. This would be consistent with, although not specific to, heightened sensory precision (Powell et al., 2020; Wurthmann et al., 2021). We further predict faster adaptation and fewer symptoms during self-directed exploration than passive viewing (Held and Freedman, 1963), reduced responses with a task-directed external focus of attention (Patel et al., 2014), and more positive affect and greater expectation updating when exposure is framed as autonomous exploration. Measures of expectation violation and cognitive immunization informed by ViolEx (Panitz et al., 2021) could also help test whether these processes predict expectation updating during vestibular rehabilitation.

We anticipate future work to design and implement empirical protocols—including qualitative, experimental, and computational approaches—capable of evaluating and refining this proposal, and we encourage collaboration across the neuroscience, vestibular, clinical, and philosophy of mind communities that this framework seeks to bring into productive dialog.

## Data Availability

The original contributions presented in the study are included in the article/supplementary material, further inquiries can be directed to the corresponding author.

## Footnotes

1 While there are established criteria for PPPD and other conditions that characterize persistent dizziness (e.g., vestibular migraine, Mal de Débarquement syndrome (MdDS), we intentionally use the broad term “persistent dizziness,” asserting that under our particular formulation, there is more in common among individuals who are living with persistent dizziness than distinguishes them. Moreover, there is considerable overlap between diagnoses and the realization that common manifestations (i.e., vestibular migraine and PPPD) exist on a continuum rather than discrete categories (Tarnutzer and Kaski, 2023; Kaski et al., 2025).

2 For a formal theoretical perspective on neural representation informed by active inference, the reader is referred to Pezzulo et al. (2024a).

3 Surprise (or surprisal) reflects observations that are uncharacteristic of an organism, based on the kind of thing that it is (or presumes to be). The imperative of minimizing surprise, on average, is equivalent to avoiding states that would produce surprising observations. Mathematically, surprise is −log p(o), which quantifies how uncharacteristic an observation is for the organism under its (phenotypic) model. Since calculating surprise is mathematically intractable, given that the organism does not have direct access to all possible observations, free energy serves as a tractable approximation (technically an upper bound on surprise). For background reading, please see Smith et al., 2022a; Parr et al., 2022).

4 Formally, a Markov Blanket. Briefly, the Markov Blanket is a statistical boundary and interface, comprised of sensory and active (blanket) states, between the “thing” (or organism) of interest and everything else. For additional reading, please see Friston (2019).

5 OPTIMAL (optimizing performance through intrinsic motivation and attention for learning), Wulf and Lewthwaite (2016). See section: “Compatibility of Active Inference with contemporary motor learning theory: OPTIMAL figure.
